# Substantial Variation in Decision Making to Perform Subacromial Decompression Surgery for Subacromial Pain Syndrome Between Orthopaedic Shoulder Surgeons for Identical Clinical Scenarios: A Case-Vignette Study

**DOI:** 10.1016/j.asmr.2023.100819

**Published:** 2023-11-11

**Authors:** Timon H. Geurkink, Perla J. Marang-van de Mheen, Jochem Nagels, Ronald N. Wessel, Rudolf W. Poolman, Rob G.H.H. Nelissen, Leti van Bodegom-Vos

**Affiliations:** a Department of Orthopaedics, Leiden University Medical Centre, Leiden, The Netherlands; b Department of Biomedical Data Sciences, Medical Decision Making, Leiden University Medical Center, Leiden, The Netherlands; c Department of Orthopaedics, Sint Antonius Hospital, Nieuwegein, The Netherlands

## Abstract

**Purpose:**

To provide further insight into the variation in decision making to perform subacromial decompression (SAD) surgery in patients with subacromial pain syndrome (SAPS) and its influencing factors.

**Methods:**

Between November 2021 and February 2022, we invited 202 Dutch Shoulder and Elbow Society members to participate in a cross-sectional Web-based survey including 4 clinical scenarios of SAPS patients. Scenarios varied in patient characteristics, clinical presentation, and other contextual factors. For each scenario, respondents were asked (1) to indicate whether they would perform SAD surgery, (2) to indicate the probability of benefit of SAD surgery (i.e., pain reduction), (3) to indicate the probability of harm (i.e., complications), and (4) to rank the 5 most important factors influencing their treatment decision.

**Results:**

A total of 78 respondents (39%) participated. The percentage of respondents who would perform SAD surgery ranged from 4% to 25% among scenarios. The median probability of perceived benefit ranged between 70% and 79% across scenarios for respondents indicating to perform surgery compared with 15% to 29% for those indicating not to perform surgery. The difference in the median probability of perceived harm ranged from 3% to 9% for those indicating to perform surgery compared with 8% to 13% for those indicating not to perform surgery. Surgeons who would perform surgery mainly reported patient-related factors (e.g., complaint duration and response to physical therapy) as the most important factors to perform SAD surgery, whereas surgeons who would not perform surgery mainly reported guideline-related factors.

**Conclusions:**

Overall, Dutch orthopaedic shoulder surgeons are reluctant to perform SAD surgery in SAPS patients. There is substantial variation among orthopaedic surgeons regarding decisions to perform SAD surgery for SAPS even when evaluating identical scenarios, where particularly the perceived benefit of surgery differed between those who would perform surgery and those who would not. Surgeons who would not perform SAD surgery mainly referred to guideline-related factors as influential factors for their decision, whereas those who would perform SAD surgery considered patient-related factors more important.

**Clinical Relevance:**

There is substantial variation in decision making to perform SAD surgery for SAPS between individual orthopaedic surgeons for identical case scenarios.

Subacromial pain syndrome (SAPS) is the most frequent diagnosis given to patients presenting with shoulder pain.^1^^,^^2^ Most SAPS patients are treated nonsurgically (e.g., glucocorticoid injections and physical therapy), but subacromial decompression (SAD) surgery can be performed when patients are not responding to nonsurgical treatment.^3^^,^^4^ SAD surgery is intended to reduce pain and improve shoulder function, but adverse effects may also occur (e.g., no pain reduction, infection, thromboembolism, or frozen shoulder).^2^^,^^5^ When considering surgery, orthopaedic surgeons must therefore carefully weigh the potential benefits of surgery against its potential harms.^6^

Recent high-quality experimental studies have found that SAD surgery provides no significant improvement in pain or functionality in SAPS patients when compared with placebo surgery or nonsurgical management^4^^,^^7^, ^8^, ^9^ whereas it still carries a risk of harm to patients. On the basis of these studies, a panel assembled by the *British Medical Journal* formulated a strong recommendation against SAD surgery for SAPS^2^ and it is considered “low-value care”—a term referring to procedures with little or no benefit or more potential harm than benefit to patients. Nevertheless, SAD surgery is still frequently performed. In the United Kingdom, the United States, and Australia, increasing trends in SAD surgery for SAPS have even been reported.^10^, ^11^, ^12^ Moreover, in the Netherlands, approximately 10,000 SAPS patients underwent SAD surgery in 2016.^13^ Consequently, several initiatives have been launched to further reduce the use of SAD surgery for SAPS worldwide. In the Netherlands, activities such as clinical guideline changes are undertaken to reduce the use of SAD surgery, and in 2020, there was a withdrawal of reimbursement through a policy change (i.e., active disinvestment) by one of the large health care insurers.

To be effective, such initiatives to reduce low-value care procedures should address factors influencing surgeons' decisions to perform surgery. Previous studies showed large variation between surgeons in their clinical decision making to perform surgery, but little is known about the factors that contribute to this variation.^14^, ^15^, ^16^ These factors include differences in patient characteristics, surgeon characteristics, surgeons’ perception of benefit and/or harm of surgical intervention, and surgeons’ knowledge and interpretation of guidelines and financial constraints.^6^^,^^15^^,^^17^ This study aimed to provide further insight into the variation in decision making to perform SAD surgery in patients with SAPS and its influencing factors. Our hypothesis was that there would be substantial variation in clinical decision making between individual orthopaedic surgeons.

## Methods

### Study Design

Between November 2021 and February 2022, we conducted a cross-sectional Web-based survey including 4 clinical scenarios among Dutch orthopaedic shoulder surgeons to examine the variation in clinical decision making to perform SAD surgery for SAPS. The study protocol (No. N20.127) was presented to the Medical Ethical Committee of Leiden University Medical Center (METC-LDD), which waived the need for ethical approval under Dutch law. All results are reported according to the Checklist for Reporting of Survey Studies (CROSS).^18^

### Setting

From January 2020 onward, 1 of the 4 largest Dutch health care insurers launched an active disinvestment initiative for SAD surgery in SAPS patients. This specific health care insurer decided to partially withdraw reimbursement for this procedure by contracting 30% fewer procedures than the preceding year in each hospital, based on (inter)national guidelines. This active disinvestment strategy was examined within the survey as one of the possible factors influencing clinical decision making of orthopaedic surgeons regarding surgical treatment of SAPS.

### Study Population

All 202 members of the Dutch Shoulder and Elbow Society (DSES) were invited to participate in the survey, which was sent on November 30, 2021. Members of the DSES are either orthopaedic shoulder surgeons or orthopaedic residents with a specific interest in shoulder surgery. At the time of our study, approximately 40 to 50 members were actively participating in DSES meetings. All members received a link for the survey by e-mail from the DSES. Two reminder e-mails were sent to all members after 3 weeks and 6 weeks, respectively. Eligible participants were orthopaedic surgeons and orthopaedic surgery residents who, on average, treated at least 1 SAPS patient per month. To prevent multiple submissions by 1 respondent, the survey could only be filled in once for every unique IP address. Participation was voluntary and anonymous. Participants were asked to further disseminate the survey to colleagues involved in treating SAPS patients.

### Survey Development

**Fig 1:**
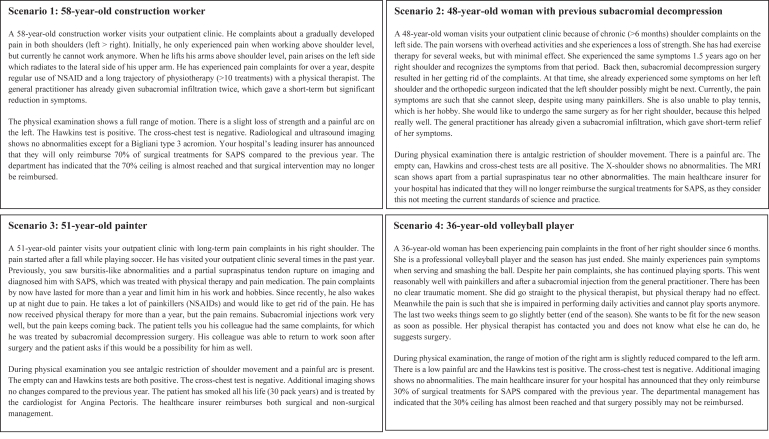
Description of clinical scenarios included in study. (MRI, magnetic resonance imaging; NSAID, nonsteroidal anti-inflammatory drug; SAPS, subacromial pain syndrome.)

Qualtrics software (Qualtrics, Provo, UT) was used to develop the survey and to perform data collection. A pilot study was carried out among 15 individuals (i.e., orthopaedic surgeons, residents, and researchers) to test the survey. The first part of the survey requested demographic information, including age, sex, current function (i.e., orthopaedic surgeon or resident), area of interest within orthopaedics, type of hospital (i.e., academic teaching hospital, non-academic teaching hospital, non-academic non-teaching hospital, or independent treatment center), and number of SAPS patients seen per month. The second part of the survey consisted of 4 hypothetical but realistic clinical scenarios regarding the treatment of an SAPS patient, each followed by 4 questions (described later; Fig 1). The last part of the survey investigated the awareness and attitude of the respondents toward the active disinvestment strategy by the health care insurer described earlier by use of 7-point Likert scales. The translated survey can be found in Appendix Figure 1.

### Clinical Scenarios

Four hypothetical clinical scenarios describing SAPS patients were developed to study the variation in clinical decision making to perform SAD surgery (Fig 1). The clinical scenarios consisted of a short paragraph and varied regarding patient characteristics, clinical presentation, outcomes of imaging tests, and other contextual factors (e.g., reimbursement status of SAD surgery). The clinical scenarios were developed by multiple orthopaedic surgeons (J.N., R.N.W., R.W.P., and R.G.H.H.N.) to ensure these were realistic for clinical cases seen in orthopaedic practice, but they were deliberately created such that there may be variation in decision making regarding whether to perform surgery or not.

Four questions accompanied each clinical scenario. The first question explored the decision of whether or not to perform SAD surgery in the patient described in the clinical scenario. The second and third questions queried the probabilities of perceived benefit (i.e., pain reduction) and harm (i.e., complications) of SAD surgery on a scale from 0% to 100%. Finally, respondents were asked to select and rank the 5 most important factors affecting their clinical decision making to perform SAD surgery or not. These factors could be selected from a predefined list (Appendix Fig 1) and included patient-related factors (e.g., characteristics of patients and their clinical presentation), guideline-related factors such as whether surgical treatment was indicated, and other contextual factors such as the reimbursement status of SAD surgery in the hospital where the patient was treated. Partially filled-in surveys were included in the analysis if at least 1 clinical scenario was completed.

### Statistical Analysis

Parametric continuous data were described using means, standard deviations, and 95% confidence intervals, whereas nonparametric data were expressed as medians and interquartile ranges (IQRs). Numbers and percentages were used to present categorical data. First, the proportion of respondents who decided to perform SAD surgery was calculated for each clinical scenario to indicate the variation in clinical decision making between scenarios. We then explored the association between respondent characteristics (i.e., age [per year], sex [female vs male], function [orthopaedic surgeon vs resident], years of experience as an orthopaedic surgeon and resident combined, type of hospital [teaching vs non-teaching], and number of SAPS patients seen per month) and the decision to perform surgery or not across all clinical scenarios using univariate logistic regression analysis with generalized estimating equations to adjust for clustering of scenarios within respondents. Factors with *P* < .20 were included in multivariate analysis to assess their independent effects. Because orthopaedic residents will inherently have less experience, which may affect their decision making, a sensitivity analysis was performed in which responses from residents were excluded.

Second, we evaluated whether the decision to perform SAD surgery or not was influenced by the perceived probabilities of benefit and harm for each of the scenarios. This was performed using a logistic model that included the logarithmically transformed benefit-harm (BH) ratio (i.e., the probability of perceived benefit divided by the probability of perceived harm) as an independent variable to predict the probability of surgery and no surgery for each respondent per clinical scenario. These predicted probabilities were plotted against the BH ratio. Using these plots, we identified the break-even point, that is, the value of the BH ratio at which the predicted probabilities of performing SAD surgery and performing no SAD surgery were equal. Assuming that surgeons decide to operate when the perceived benefits outweigh the perceived harms, the probability of performing surgery can be expected to exceed the probability of not performing surgery when the BH ratio is greater than 1.

Finally, we used descriptive statistics to evaluate which factors were most important for the decision to perform SAD surgery or not, as well as the perceived effect of the active disinvestment strategy on clinical decision making. Stata software (version 17.1; StataCorp, College Station, TX) and SPSS software (version 20.0; IBM, Armonk, NY) were used for analysis. The level of significance was established at *P* < .05.

## Results

Of 202 invited members of the DSES, 78 (39%) participated in the study. Fourteen respondents (18%) did not complete the first clinical scenario, thus leaving 64 respondents (82%) for analysis. Of these, 57 (89%) completed all questions. The respondents who did not complete the first clinical scenario did not differ in demographic characteristics from the group included in the analysis (data not shown). Among respondents, 52 (81%) were orthopaedic surgeons and 12 (19%) were orthopaedic residents. Respondents had a mean age of 45 years (standard deviation, 9.4 years), and most (80%) were men. Most respondents (55%) worked in a non-academic teaching hospital. Table 1 shows the baseline characteristics of the respondents.

**Table 1:** Characteristics of Respondents Who Participated in Study

	Orthopaedic Surgeons	Orthopaedic Residents
n (%)	52 (81)	12 (19)
Mean age (SD), yr	48 (7.7)	32 (2.9)
% Female sex	15	33
Mean experience (SD), yr	12 (7.1)	NA
Median year of residency (IQR)	NA	5 (4-5)
Area of interest, %		
Shoulder	98	58
Elbow	58	25
Wrist and hand	27	0
Spine	0	0
Hip	14	33
Knee	31	67
Ankle and feet	4	8
Sport	29	25
Traumatology	39	58
Pediatrics	6	0
Type of hospital, %		
General		
Teaching	50	75
Non-teaching	31	8
Academic	2	17
Private	10	0
Other	8	0
Median No. of SAPS patients per month (IQR)	48 (25-74)	10 (3-28)

IQR, interquartile range; NA, not applicable; SAPS, subacromial pain syndrome; SD, standard deviation.

### Decision to Perform Surgery

The decision to perform surgery varied among the 4 clinical scenarios. In the first clinical scenario (58-year-old construction worker), 8 respondents (13%) would perform SAD surgery. In the second (48-year-old woman with previous SAD) and third (51-year-old painter) clinical scenarios, 13 respondents (22%) and 14 respondents (25%), respectively, would perform SAD surgery, whereas only 2 respondents (4%) would perform surgery in the fourth clinical scenario (36-year-old volleyball player). None of the respondent characteristics was associated with the decision to perform SAD surgery (using *P* < .20 as the threshold; Appendix Table 1) so that multivariate analysis was not conducted. The results remained the same when the responses of the orthopaedic residents were excluded in the sensitivity analysis (Appendix Table 1).

### Perceived Benefits and Harms of Surgery

The median probability of perceived benefit across respondents varied from 15% to 36% among clinical scenarios, and the median probability of perceived harm ranged from 8% to 10%. An interesting finding was that the median probability of perceived benefit for respondents who decided to perform SAD surgery ranged from 70% to 79% among clinical scenarios compared with a range of 15% to 29% for those deciding not to perform surgery. Much smaller differences were observed in the probabilities of perceived harm, ranging from 3% to 9% among clinical scenarios for surgeons deciding to perform surgery and from 8% to 13% for those who would not perform surgery (Table 2).

**Table 2:** Probabilities of Perceived Benefit (i.e., Pain Reduction) and Harm (i.e., Complications) of Subacromial Decompression Surgery for Each Clinical Scenario, Stratified by Decision to Perform Surgery

	Clinical Scenario							
	Scenario 1: 58-Year-Old Construction Worker		Scenario 2: 48-Year-Old Woman With Previous Subacromial Decompression		Scenario 3: 51-Year-Old Painter		Scenario 4: 36-Year-Old Volleyball Player	
	No Surgery	Surgery	No Surgery	Surgery	No Surgery	Surgery	No Surgery	Surgery
n (%)	56 (87)	8 (13)	47 (78)	13 (22)	43 (75)	14 (25)	55 (96)	2 (4)
Median probability of benefit (IQR)	25 (10-50)	75 (70-88)	29 (10-50)	70 (50-81)	20 (9-39)	79 (50-81)	15 (6-39)	71 (70-71)
Median probability of harm (IQR)	9 (4-17)	9 (7-10)	10 (5-19)	5 (4-12)	13 (8-20)	8 (4-14)	8 (3-15)	3 (1-4)

IQR, interquartile range.

**Fig 2:**
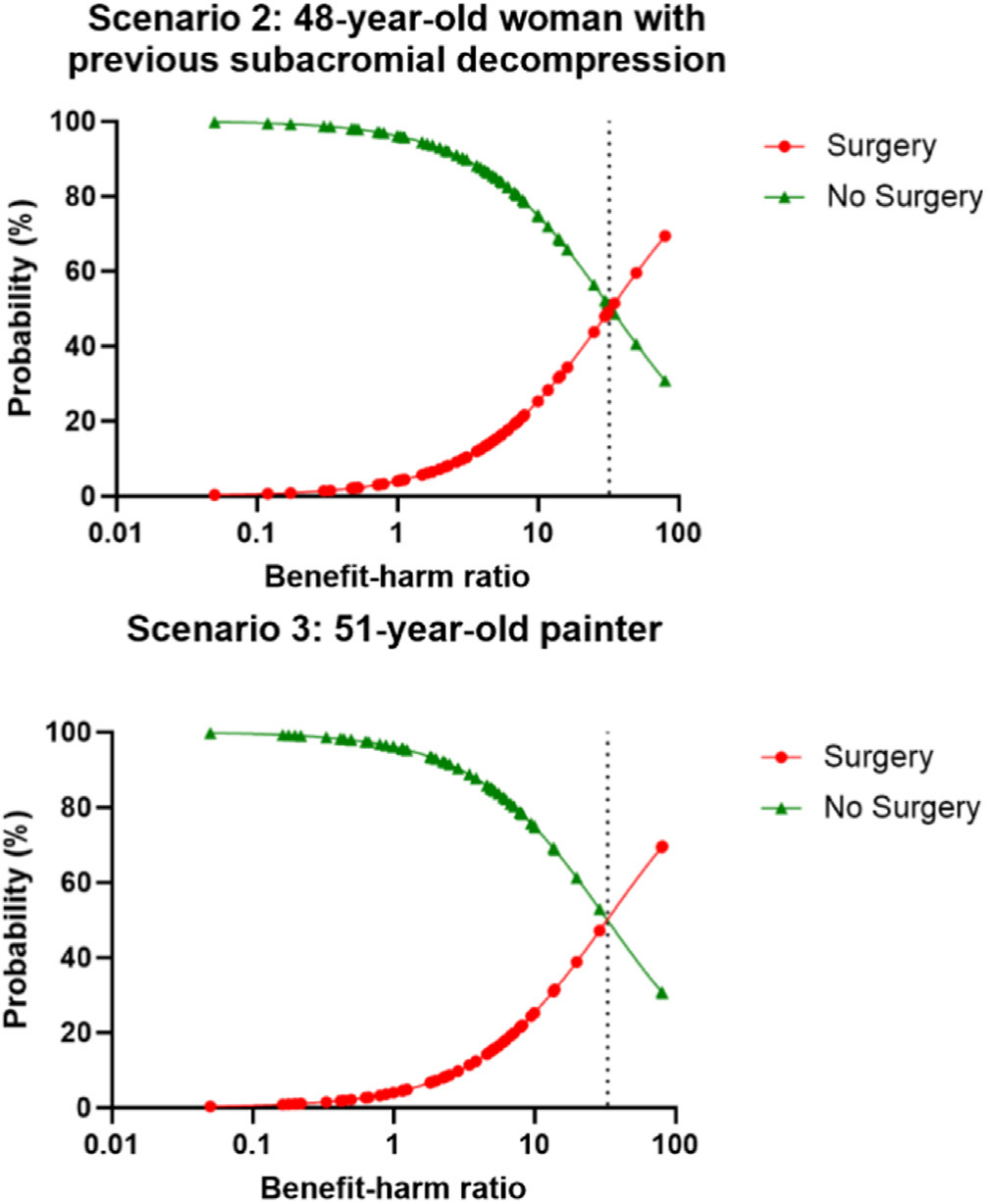
Predicted probability of subacromial decompression (SAD) surgery and no SAD surgery versus benefit-harm ratio for 2 clinical scenarios. The dotted line represents the break-even point (i.e., the value of the benefit-harm ratio at which the predicted probability of performing SAD surgery and the predicted probability of not performing SAD surgery were equal [50%]): 32 for clinical scenario 2 (48-year-old woman with previous subacromial decompression) and 33 for clinical scenario 3 (51-year-old painter).

The BH ratio was significantly associated with the decision to perform surgery (odds ratio, 8.2; 95% confidence interval, 3.7-18.1; *P* < .001). The break-even point of the BH ratio (i.e., the value of the BH ratio at which the predicted probabilities of surgery and no surgery were equal [50%]) was 32 and 33 for scenario 2 (48-year-old woman with previous SAD) and scenario 3 (51-year-old painter), respectively (Figs 1 and 2). Thus, only when the perceived benefit is 32 or 33 times higher than the perceived harm will the predicted probability of receiving SAD surgery for SAPS exceed 50%. The break-even point was not calculated for the other scenarios given that few surgeons would perform surgery in these scenarios.

### Factors Influencing Decision to Perform Surgery

Table 3 provides a list of the factors ranked as most important by respondents in their decision to perform surgery or not for each scenario. Among the respondents who decided to perform surgery, the duration of complaints, effectiveness of subacromial infiltration, outcomes of imaging tests, and response to targeted physical therapy were most frequently reported as the factors that were the most important for the decision to perform SAD surgery. In contrast, among respondents who decided not to perform surgery “surgical treatment not indicated,” outcomes of imaging tests, and “non-surgical treatment better” were most frequently reported as the factors important for the decision. The presence or absence of reimbursement for SAD surgery (i.e., reimbursement status) was scarcely reported as an important factor (range, 3%-9% among clinical scenarios). Only 51% of the respondents were familiar with the active disinvestment strategy for SAD surgery by the health care insurer, of whom 18% could name the specific insurer implementing this strategy. Respondents who decided to perform SAD surgery for at least 1 clinical scenario reported that there was insufficient evidence to stop reimbursement for SAD surgery in SAPS patients (median, 3 [IQR, 2-3], on a Likert scale from 1 [absolutely insufficient evidence] to 7 [absolutely sufficient evidence]), whereas surgeons deciding not to perform surgery believed that there was sufficient evidence (median, 5 [IQR, 3-6]).

**Table 3:** Most Important Factors for Decision to Perform Surgery or Not for Each Clinical Scenario, Stratified by Decision to Perform Surgery

Treatment Decision	Clinical Scenario			
	Scenario 1: 58-Year-Old Construction Worker	Scenario 2: 48-Year-Old Woman With Previous Subacromial Decompression	Scenario 3: 51-Year-Old Painter	Scenario 4: 36-Year-Old Volleyball Player
Subacromial decompression	n = 8 (13%) >1 yr of complaints (n = 6) Shape of acromion (Bigliani type 3) (n = 6) Reduction of complaints after subacromial infiltration (n = 5) Unable to work (n = 5)	n = 13 (22%) Imaging: partial supraspinatus rupture and no signs of bursitis (n = 10) >6 mo of complaints (n = 6) Reduction of complaints after subacromial infiltration (n = 6) Specific tests: findings of empty can, Hawkins test, and cross-chest test all positive (n = 5)	n = 14 (25%) >1 yr of complaints (n = 11) Imaging: bursitis-like abnormalities and partial supraspinatus tendon rupture (n = 8) Progressive complaints (n = 6) No effect of exercise therapy on complaints (n = 5)	n = 2 (4%)∗ Professional volleyball player (n = 2) No effect of physical therapy on complaints (n = 2)
No subacromial decompression	n = 56 (87%) Surgical treatment not indicated (n = 41) Imaging: no abnormalities on ultrasound or imaging (n = 31) Nonsurgical treatment better (n = 30) Complaints bilateral (n = 15)	n = 47 (78%) Surgical treatment not indicated (n = 36) Imaging: partial supraspinatus rupture and no signs of bursitis (n = 26) Nonsurgical treatment better (n = 23) Female aged 48 yr (n = 11)	n = 43 (75%) Surgical treatment not indicated (n = 25) Imaging: bursitis-like abnormalities and partial supraspinatus tendon rupture (n = 24) Comorbidities of patient (n = 19) Nonsurgical treatment better (n = 17)	n = 55 (96%) Surgical treatment not indicated (n = 39) Imaging: no abnormalities (n = 35) Nonsurgical treatment better (n = 27) Female aged 36 yr (n = 25)

∗ Only the 2 most important factors are shown owing to the low fraction of surgeons deciding to perform subacromial decompression surgery in this clinical scenario.

## Discussion

Consistently with our hypothesis, this study showed that there was substantial variation in the decision making to perform surgery for SAPS between orthopaedic shoulder surgeons. Overall, the respondents were reluctant to perform SAD surgery as shown by the high break-even points indicating that the perceived benefit of SAD surgery had to substantially outweigh the harm before most respondents decided to perform surgery. The decision to perform SAD surgery seemed to depend particularly on differences in the perceived benefits of surgery rather than differences in the perceived harms. Additionally, surgeons who decided to perform SAD surgery mainly reported patient-related factors to be among the most important factors, whereas surgeons who decided not to perform surgery mainly reported factors related to current clinical guidelines.

The overall reluctance to perform SAD surgery for SAPS in this study is in line with current evidence, the Dutch national guideline of the Netherlands Orthopaedic Association, and the clinical practice guideline recommendation by the *British Medical Journal* panel.^2^^,^^4^^,^^7^^,^^19^ Consistently with this, Veen et al.^13^ previously reported a decreasing trend in the use of SAD surgery for SAPS in the Netherlands but still approximately 7% of the patients with an SAPS diagnosis underwent surgery in 2016. Decreasing trends have also been reported in various other countries such as Scotland and Finland,^20^^,^^21^ but increasing trends have been described for Australia, the United Kingdom, and the United States.^10^^,^^11^^,^^22^ The previously described conflicting trends highlight the need for studies exploring factors that might drive decisions to perform SAD surgery for SAPS despite the presence of high-quality evidence showing no benefit.

This study explored the variation in clinical decision making to perform surgery for a low-value care procedure such as SAPS by using clinical case vignettes. Previous studies examined the variation in clinical decision making for various other surgical interventions (e.g., rotator cuff repair and gastrointestinal surgical procedures).^6^^,^^17^^,^^23^ These studies not only showed substantial variation in the clinical decision making to perform surgery between surgeons^17^^,^^24^^,^^25^ but also showed that this variation occurred within surgeons over time when the scenarios remained identical.^23^ This finding suggests that the decision to perform surgery may depend on the subjective clinical judgment of a surgeon at a specific time point, but it is unknown what factors may have influenced the change in a surgeon’s judgment over time. Sacks et al.^6^ studied how general surgeons’ judgment regarding the likelihood of benefit and harm of surgery influenced their decision to perform surgery. They reported that surgeons were more likely to perform surgery when their perceived benefit of surgery was high and their perceived likelihood of harm was low. The results of our study add to this literature that variation in the decision to perform surgery mainly seems to result from differences in perceived benefit rather than harm. Similar findings were reported by a nonsurgical study that evaluated the variation in transfusion decisions (i.e., red blood cell transfusion) within the intensive care unit.^26^

It is unclear which factors drive differences in the perceived benefit of surgery. Dunn et al.^17^ found that orthopaedic surgeons who performed a high volume of rotator cuff repair procedures had higher expectations of the surgical intervention than those who performed a low volume of procedures. Therefore, it might reflect that these surgeons value their own experience higher than evidence from guidelines and the literature.^27^^,^^28^ However, inadequate judgment of perceived benefit may also result from cognitive biases in decision making, such as the tendency of clinicians to overestimate the benefits and underestimate the harms of interventions (i.e., impact bias).^29^ Training surgeons to make them more aware of the influence of cognitive bias on their decision making may help to improve this.^30^ It is also possible that surgeons first decide to perform surgery in a particular clinical scenario and subsequently match their assessment of potential benefit and harm with their decision,^31^ which would suggest that we need to study factors influencing the decision to perform surgery rather than differences in perceived benefit. Finally, it is hypothesized that the variation in perceived benefit might be the result of different weighing of factors in the clinical scenarios,^26^ which is consistent with our results showing differences in factors reported as most important between surgeons who would perform surgery and those who would not perform surgery.

Previous literature has shown that factors such as patient characteristics, scientific evidence, clinical guidelines, and financial constraints are important factors in the decision-making process regarding surgery.^32^ In this study, we found that surgeons who would not perform surgery mainly reported factors related to current clinical guideline recommendations whereas surgeons who would perform SAD surgery reported the importance of clinical benefits. Wright et al.^33^ have previously proposed that the paucity of evidence, the controversy around evidence, and a lack of awareness or acceptance of evidence may cause differences in the interpretation and acceptance of clinical guidelines and evidence. Consistently with this, we found that respondents who would perform SAD surgery for at least 1 clinical scenario reported that there was insufficient evidence to stop reimbursement for SAD surgery for SAPS whereas surgeons who would not perform surgery indicated that there was sufficient evidence to justify such an initiative. It is interesting to note that orthopaedic surgeons more often decided to perform surgery in the clinical scenarios in which the patients had a partial supraspinatus tear (i.e., scenarios 2 and 3). A possible explanation may be that patients with a high-grade partial-thickness tear were excluded in previous randomized trials, which surgeons might have taken as an indication that these patients might still benefit from SAD surgery.^19^ Additionally, surgeons who would perform SAD surgery weighed patient-related factors more heavily in their decision, indicating a different interpretation of the guidelines or indicating that they might find it difficult to abstain from surgical treatment in case of (long-lasting) patient complaints.^34^ The latter would suggest that action bias,^35^ that is, the general preference for active over passive treatment in clinical decision making, might also play a role. Unfortunately, there is no consensus on what alternative treatment is best for SAPS patients, leaving the clinicians with uncertainty, which might further contribute to action bias.^36^

Knowing when to perform surgery is considered a critical skill for a surgeon.^6^^,^^28^ Our study findings highlight the complexity of reducing the use of a low-value care procedure in daily practice even if based on strong recommendations. Ultimately, decisions to perform surgery (or not) are not only based on objective evidence but also based on subjective clinical judgment, which in turn depends on surgeons’ personal experiences and beliefs. Changing clinician behavior is therefore considered an extremely complex process.^37^ Most interventions that aim to reduce low-value care, such as guideline changes or the withdrawal of reimbursement, address only objective evidence but not the subjective clinical judgment of surgeons. Therefore, it is unlikely that such solitary interventions will be effective. The results of this study highlight the necessity for multifaceted interventions that address objective evidence but also target surgeons' personal beliefs and perceptions because these will be more likely to have an effect.

The strength of this study is that we used the same clinical scenarios for every respondent to study the variation in clinical decision making for SAD surgery and its influencing factors. This allowed for a better evaluation of differences in decision making between orthopaedic surgeons rather than these decisions being influenced by differences in (complexity of) patients. Whereas most studies have only investigated the influence of patient-related factors,^6^^,^^26^ we also included guideline-related factors and contextual factors in the clinical scenarios. Doing so provides a more complete understanding of the factors contributing to variation in surgical decision making. Furthermore, respondents could fill out the survey anonymously, which likely improves respondents’ willingness to give honest rather than socially desirable answers.

### Limitations

Limitations of our study include the low response rate (39%). There will be inactive members in any professional society, who will not be likely to respond to a questionnaire. However, it is also possible that members had an interest in shoulder and elbow surgery but did not frequently see and treat SAPS patients and therefore did not respond, given that the survey stipulated that at least 1 SAPS patient should be treated per month to participate. Because only 40 to 50 DSES members were actively participating in meetings and there were 64 respondents who participated in this study, it seems that our study is likely representative of surgeons frequently performing shoulder surgery or treating sufficient numbers of SAPS patients. Second, common method bias may have influenced our results because both the independent and dependent variables were part of the same questionnaire.^38^ To reduce the likelihood of this occurring, we varied the factors that could be chosen across scenarios, varied the question types, and used different wording within the questions that included a Likert scale. Finally, because we conducted this survey among orthopaedic surgeons and residents in the Netherlands, the results of our study may not be generalizable to other countries, given that other factors influencing the decision to perform SAD surgery may be more relevant in other settings. However, the overarching finding of variation in clinical decision making being particularly influenced by differences in perceived benefit and not perceived harm to patients will likely also apply to other countries given the results of other studies.^26^

## Conclusions

Overall, Dutch orthopaedic shoulder surgeons are reluctant to perform SAD surgery in SAPS patients. There is substantial variation among orthopaedic surgeons regarding decisions to perform SAD surgery for SAPS even when evaluating identical scenarios, where particularly the perceived benefit of surgery differed between those who would perform surgery and those who would not. Surgeons who would not perform SAD surgery mainly referred to guideline-related factors as influential factors for their decision, whereas those who would perform SAD surgery considered patient-related factors more important.

## Disclosure

The authors report the following potential conflicts of interest or sources of funding: This work was supported by a grant (No. 80-83920-98-803) from ZonMW, the Dutch Organization for 10.13039/100005622Health Research and Development. ZonMW did not influence the study in any way or the writing of the manuscript. T.H.G. reports grant support from ZonMW. R.G.H.H.N. reports grant support from ZonMW. L.v.B-V. reports grant support from ZonMW. All other authors declare that they have no known competing financial interests or personal relationships that could have appeared to influence the work reported in this paper. Full ICMJE author disclosure forms are available for this article online, as supplementary material.

## Supplementary Data

ICMJE author disclosure forms

Appendix Figure 1 Translated survey.

Appendix Table 1 Influence of Respondents’ Characteristics on Decision to Perform Surgery Across All Scenarios, Adjusted for Clustering Within Respondents and Sensitivity Analysis

## References

[1] Mitchell C., Adebajo A., Hay E., Carr A. (2005). Shoulder pain: Diagnosis and management in primary care. BMJ.

[2] Vandvik P.O., Lahdeoja T., Ardern C. (2019). Subacromial decompression surgery for adults with shoulder pain: A clinical practice guideline. BMJ.

[3] Cummins C.A., Sasso L.M., Nicholson D. (2009). Impingement syndrome: Temporal outcomes of nonoperative treatment. J Shoulder Elbow Surg.

[4] Kolk A., Thomassen B.J.W., Hund H. (2017). Does acromioplasty result in favorable clinical and radiologic outcomes in the management of chronic subacromial pain syndrome? A double-blinded randomized clinical trial with 9 to 14 years' follow-up. J Shoulder Elbow Surg.

[5] Lähdeoja T., Karjalainen T., Jokihaara J. (2020). Subacromial decompression surgery for adults with shoulder pain: A systematic review with meta-analysis. Br J Sports Med.

[6] Sacks G.D., Dawes A.J., Ettner S.L. (2016). Surgeon perception of risk and benefit in the decision to operate. Ann Surg.

[7] Beard D.J., Rees J.L., Cook J.A. (2018). Arthroscopic subacromial decompression for subacromial shoulder pain (CSAW): A multicentre, pragmatic, parallel group, placebo-controlled, three-group, randomised surgical trial. Lancet.

[8] Paavola M., Kanto K., Ranstam J. (2021). Subacromial decompression versus diagnostic arthroscopy for shoulder impingement: A 5-year follow-up of a randomised, placebo surgery controlled clinical trial. Br J Sports Med.

[9] Henkus H.E., de Witte P.B., Nelissen R.G., Brand R., van Arkel E.R. (2009). Bursectomy compared with acromioplasty in the management of subacromial impingement syndrome: A prospective randomised study. J Bone Joint Surg Br.

[10] Jones T., Carr A.J., Beard D. (2019). Longitudinal study of use and cost of subacromial decompression surgery: The need for effective evaluation of surgical procedures to prevent overtreatment and wasted resources. BMJ Open.

[11] Thorpe A., Hurworth M., O'Sullivan P., Mitchell T., Smith A. (2016). Rising trends in surgery for rotator cuff disease in Western Australia. ANZ J Surg.

[12] Vitale M.A., Arons R.R., Hurwitz S., Ahmad C.S., Levine W.N. (2010). The rising incidence of acromioplasty. J Bone Joint Surg Am.

[13] Veen E.J.D., Stevens M., Koorevaar C.T., Diercks R.L. (2019). Appropriate care for orthopedic patients: Effect of implementation of the Clinical Practice Guideline for Diagnosis and Treatment of Subacromial Pain Syndrome in the Netherlands. Acta Orthop.

[14] Alderman A.K., Chung K.C., Kim H.M., Fox D.A., Ubel P.A. (2003). Effectiveness of rheumatoid hand surgery: Contrasting perceptions of hand surgeons and rheumatologists. J Hand Surg.

[15] Wilson N.P., Wilson F.P., Neuman M. (2013). Determinants of surgical decision making: A national survey. Am J Surg.

[16] Birkmeyer J.D., Reames B.N., McCulloch P., Carr A.J., Campbell W.B., Wennberg J.E. (2013). Understanding of regional variation in the use of surgery. Lancet.

[17] Dunn W.R., Schackman B.R., Walsh C. (2005). Variation in orthopaedic surgeons' perceptions about the indications for rotator cuff surgery. J Bone Joint Surg Am.

[18] Sharma A., Minh Duc N.T., Luu Lam Thang T. (2021). A Consensus-Based Checklist for Reporting of Survey Studies (CROSS). J Gen Intern Med.

[19] Paavola M., Malmivaara A., Taimela S. (2018). Subacromial decompression versus diagnostic arthroscopy for shoulder impingement: Randomised, placebo surgery controlled clinical trial. BMJ.

[20] Paloneva J., Lepola V., Karppinen J., Ylinen J., Aarimaa V., Mattila V.M. (2015). Declining incidence of acromioplasty in Finland. Acta Orthop.

[21] Jenkins P.J., Stirling P.H.C., Ireland J., Elias-Jones C., Brooksbank A.J. (2020). The changing incidence of arthroscopic subacromial decompression in Scotland. Bone Joint J.

[22] Iyengar J.J., Samagh S.P., Schairer W., Singh G., Valone F.H., Feeley B.T. (2014). Current trends in rotator cuff repair: Surgical technique, setting, and cost. Arthroscopy.

[23] Rutkow I.M. (1982). Surgical decision making. The reproducibility of clinical judgement. Arch Surg.

[24] Rutkow I.M., Gittelsohn A.M., Zuidema G.D. (1979). Surgical decision making. The reliability of clinical judgment. Ann Surg.

[25] Rutkow I.M., Starfield B.H. (1984). Surgical decision making and operative rates. Arch Surg.

[26] Kranenburg F.J., Willems S.A., Le Cessie S., Marang-van de Mheen P.J., van der Bom J.G., Arbous M.S. (2018). Variation in red cell transfusion decisions in the intensive care unit—A nationwide survey in the Netherlands. Vox Sang.

[27] Rietbergen T., Diercks R., Wel I. (2019). Preferences and beliefs of Dutch orthopaedic surgeons and patients reduce the implementation of “Choosing Wisely” recommendations in degenerative knee disease. Knee Surg Sports Traumatol Arthrosc.

[28] Meunier A., Posadzy K., Tinghög G., Aspenberg P. (2017). Risk preferences and attitudes to surgery in decision making. Acta Orthop.

[29] Scott I.A., Soon J., Elshaug A.G., Lindner R. (2017). Countering cognitive biases in minimising low value care. Med J Aust.

[30] Janssen S.J., Teunis T., Ring D., Parisien R.C. (2021). Cognitive biases in orthopaedic surgery. J Am Acad Orthop Surg.

[31] Hamminga J.F., Marang-van de Mheen P.J. (2017). How does a surgeon decide that surgery is the best treatment option?. Ned Tijdschr Geneeskd.

[32] Gunaratnam C., Bernstein M. (2018). Factors affecting surgical decision-making—A qualitative study. Rambam Maimonides Med J.

[33] Wright J.G., Coyte P., Hawker G. (1995). Variation in orthopedic surgeons' perceptions of the indications for and outcomes of knee replacement. CMAJ.

[34] Buchbinder R., Karjalainen T.V., Gorelik A. (2022). Editorial commentary: Arthroscopic treatment should no longer be offered to people with subacromial impingement. Arthroscopy.

[35] Ayanian J.Z., Berwick D.M. (1991). Do physicians have a bias toward action? A classic study revisited. Med Decis Making.

[36] van Bodegom-Vos L., Marang-van de Mheen P. (2022). Reducing low-value care: Uncertainty as crucial cross-cutting theme: Comment on "Key factors that promote low-value care: Views of experts from the United States, Canada, and the Netherlands.". Int J Health Policy Manag.

[37] Godin G., Bélanger-Gravel A., Eccles M., Grimshaw J. (2008). Healthcare professionals' intentions and behaviours: A systematic review of studies based on social cognitive theories. Implement Sci.

[38] Jordan P.J., Troth A.C. (2019). Common method bias in applied settings: The dilemma of researching in organizations. Aust J Manage.

