# Supplementary material for: Substantial Variation in Decision Making to Perform Subacromial Decompression Surgery for Subacromial Pain Syndrome Between Orthopaedic Shoulder Surgeons for Identical Clinical Scenarios: A Case-Vignette Study
Source: Arthrosc Sports Med Rehabil. 2023 Nov 11;5(6):100819. doi: 10.1016/j.asmr.2023.100819 (PMC10661501; doi:10.1016/j.asmr.2023.100819)
Supplement: Appendix Figure 1 — Translated survey. [file mmc2.docx]

**Appendix S1. Survey**

Dear Colleague,

Thank you for taking part in this survey!

This is a study* on policy measures that are used by healthcare insurers to stimulate the appropriate use of healthcare services. This survey specifically focuses on the treatment **of subacromial pain syndrome (SAPS)**.

Completion of the survey is anonymous and voluntary and takes approximately 10-15 minutes. If the survey is interrupted (intentionally or unintentionally), you can pick up where you left off later by clicking on the survey link again.

Press next to start the survey.

*This study is part of the programme “Doen of Laten” and funded by “het Citrienfonds”, an initiative of the NFU and ZonMW. This study is conducted by the Leiden University Medical Centre, Orthopaedics and Medical Decision Making departments.

**Part 1. Respondent characteristics.**

*In the first part of this survey, we ask you to answer some questions with regard to demographic variables, professional background and interests.*

- What is your gender?
  - Male
  - Female
  - Other
  - Prefer not to answer
- What is your age? (in years)
  - …..
- What is your current employment?
  - Orthopedic surgeon
    - If orthopedic surgeon:
      - How many years of experience as an orthopedic surgeon do you have?
        - …….
  - Orthopedic surgery resident, in training
    - If orthopedic surgery resident, in training.
      - In which year of residency are you in?
        - 1
        - 2
        - 3
        - 4
        - 5
        - 6
  - Orthopedic surgery resident, not in training.
  - Other, ….
- Do you have other employments in the orthopedic field? (multiple answers possible)
  - Physician educator
  - Researcher
  - Member of guideline committee
  - No other employments
  - Other, ….
- What is your area of interest within orthopedic surgery? (multiple answers possible)
  - Shoulder
  - Elbow
  - Hand/Wrist
  - Spine
  - Hip
  - Knee
  - Foot/Ankle
  - Sports
  - Pediatrics
  - Traumatology
  - Other, ……
- In which type of hospital do you work for most part of the week?
  - General hospital, teaching
  - General hospital, non-teaching
  - Independent treatment center
  - Academic hospital
  - Other, …..
- How many SAPS patients do you see on average per month (please give an estimate with regard to the pre-pandemic period)? If you do not see any SAPS patients, you will be redirected to the end of the survey.
  - ….. Patients.
    - If 0, the respondent is redirected to the end of the survey.

**Part 2. Influence of reimbursement on treatment decisions**

*The next part of the survey contains questions about the extent to which the reimbursement status of a specific treatment by the patient’ healthcare insurer influences your clinical decision-making.*

1. In clinical decision-making, to what extent do you consider whether a treatment is reimbursed by the healthcare insurer?

I do not weigh this at all in my decision I weigh this considerably in my decision

|  | 1 | 2 | 3 | 4 | 5 | 6 | 7 |
| --- | --- | --- | --- | --- | --- | --- | --- |

|  | 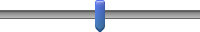 |
| --- | --- |

2. How often do you perform surgery in patients with SAPS? (please give an estimate with regard to the pre-pandemic period)?

Never Weekly

|  | 1 | 2 | 3 | 4 | 5 | 6 | 7 |
| --- | --- | --- | --- | --- | --- | --- | --- |

|  | 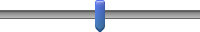 |
| --- | --- |

3. In a decision to perform subacromial decompression surgery in a patient with SAPS, to what extent do you consider whether a subacromial decompression is reimbursed by the healthcare insurer?

I do not weigh this at all in my decision I weigh this considerably in my decision

|  | 1 | 2 | 3 | 4 | 5 | 6 | 7 |
| --- | --- | --- | --- | --- | --- | --- | --- |

|  | 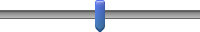 |
| --- | --- |

**Part 3. Clinical scenarios**

*In this part of the survey, we will present you with 4 clinical cases. For each case, there will be 5 questions about that specific case. Please choose the most suitable answer for every question.*

Clinical case 1

A 58-year-old construction worker visits your outpatient clinic. He complaints about a gradually developed pain in both shoulders (left > right). Initially, he only experienced pain when working above shoulder level, but currently he cannot work anymore. When he lifts his arms above shoulder level, pain arises on the left side which radiates to the lateral side of his upper arm. He has experienced pain complaints for over a year, despite regular use of NSAID and a long trajectory of physical therapy (>10 treatments) with a physical therapist. The general practitioner has already given subacromial infiltration twice, which gave a short-term but significant reduction in symptoms.

The physical examination shows a full range of motion. There is a slight loss of strength and a painful arc on the left. The Hawkins test is positive. The cross-chest test is negative. Radiological and ultrasound imaging shows no abnormalities except for a Bigliani type 3 acromion. Your hospital’s leading insurer has announced that they will only reimburse 70% of surgical treatments for SAPS compared to the previous year. The department has indicated that the 70% ceiling is almost reached and that surgical intervention may no longer be reimbursed.

1. Would you perform subacromial decompression surgery in this patient?

- Yes
- No

2. What is your estimate of the likelihood of pain reduction from subacromial decompression surgery in this patient?

|  | 0 | 10 | 20 | 30 | 40 | 50 | 60 | 70 | 80 | 90 | 100 |
| --- | --- | --- | --- | --- | --- | --- | --- | --- | --- | --- | --- |

| I estimate the likelihood of pain reduction (%) | 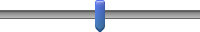 |
| --- | --- |

3. What is your estimate of the risk of complications from subacromial decompression surgery in this patient?

|  | 0 | 10 | 20 | 30 | 40 | 50 | 60 | 70 | 80 | 90 | 100 |
| --- | --- | --- | --- | --- | --- | --- | --- | --- | --- | --- | --- |

| I estimate the likelihood of complications (%) | 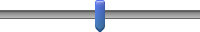 |
| --- | --- |

4. What are the most important factors influencing your decision to perform subacromial decompression surgery or not in this patient?

Choose the **most important** factors (minimum 3, maximum 5)

*- Male, 58 years
- Construction worker
- > 1 Year complaints
- Progressive complaints
- Unable to work
- Complaints bilateral
- First presentation at the outpatient clinic
- No history of trauma
- Regular use of painkillers
- Full range of motion
- Loss of strength
- Painful arc
- Specific tests: Hawkins positive, Cross-chest negative
- Shape acromion (Bigliani type 3)
- Imaging: no abnormalities on ultrasound/MRI
- Non-surgical treatment better
- Other surgery better
- Surgery possibly not reimbursed
- Surgical treatment not indicated
- No effect of physical therapy on complaints
- Reduction of complaints after subacromial infiltration
- No explicit wish of patient to have surgery*

5. Please rank the factors that you have chosen where 1 indicates the most important factor.

1. …..
2. …..
3. …..
4. …..
5. …..

Clinical case 2

A 48-year-old woman visits your outpatient clinic because of chronic (>6 months) shoulder complaints on the left side. The pain worsens with overhead activities and she experiences a loss of strength. She has had exercise therapy for several weeks, but with minimal effect. She experienced the same symptoms 1.5 years ago on her right shoulder and recognizes the symptoms from that period. Back then, subacromial decompression surgery resulted in her getting rid of the complaints. At that time, she already experienced some symptoms on her left shoulder and the orthopedic surgeon indicated that the left shoulder possibly might be next. Currently, the pain symptoms are such that she cannot sleep, despite using many painkillers. She is also unable to play tennis, which is her hobby. She would like to undergo the same surgery as for her right shoulder, because this helped really well. The general practitioner has already given a subacromial infiltration, which gave short-term relief of her symptoms.

During physical examination there is antalgic restriction of shoulder movement. There is a painful arc. The empty can, Hawkins and cross-chest tests are all positive. The X-shoulder shows no abnormalities. The MRI scan shows apart from a partial supraspinatus tear no other abnormalities. The main healthcare insurer for your hospital has indicated that they will no longer reimburse the surgical treatments for SAPS, as they consider this not meeting the current standards of science and practice.

1. Would you perform subacromial decompression surgery in this patient?

- Yes
- No

2. What is your estimate of the likelihood of pain reduction from subacromial decompression surgery in this patient?

|  | 0 | 10 | 20 | 30 | 40 | 50 | 60 | 70 | 80 | 90 | 100 |
| --- | --- | --- | --- | --- | --- | --- | --- | --- | --- | --- | --- |

| I estimate the likelihood of pain reduction (%) | 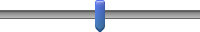 |
| --- | --- |

3. What is your estimate of the risk of complications from subacromial decompression surgery in this patient?

|  | 0 | 10 | 20 | 30 | 40 | 50 | 60 | 70 | 80 | 90 | 100 |
| --- | --- | --- | --- | --- | --- | --- | --- | --- | --- | --- | --- |

| I estimate the likelihood of complications (%) | 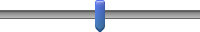 |
| --- | --- |

4. What are the most important factors influencing your decision to perform subacromial decompression surgery or not in this patient?

*Factors:*

- *Female, 48 years*
- *> 6 months complaints*
- *Progressive complaints*
- *Complaints limiting sports/hobbies/sleeping*
- *Unilateral complaints*
- *No history of trauma*
- *Uses a lot of painkillers*
- *Range of motion limited*
- *Loss of strength*
- *Painful arc*
- *Specific tests: empty can, Hawkins test and cross-chest test all positive*
- *No abnormalities on X-shoulder*
- *Imaging: partial supraspinatus rupture, no signs of bursitis*
- *Non-surgical treatment better*
- *Other surgery better*
- *Surgery not reimbursed*
- *Surgical treatment not indicated*
- *Minimal effect of physical therapy on complaints*
- *Reduction of symptoms after subacromial infiltration*
- *Previous surgery contralateral side effective*
- *Suggestion from previous surgeon*
- *Wish patient to have surgery*

5. Please rank the factors that you have chosen where 1 indicates the most important factor.

1. …..
2. …..
3. …..
4. …..
5. …..

Clinical case 3

A 51-year-old painter visits your outpatient clinic with long-term pain complaints in his right shoulder. The pain started after a fall while playing soccer. He has visited your outpatient clinic several times in the past year. Previously, you saw bursitis-like abnormalities and a partial supraspinatus tendon rupture on imaging and diagnosed him with SAPS, which was treated with physical therapy and pain medication. The pain complaints by now have lasted for more than a year and limit him in his work and hobbies. Since recently, he also wakes up at night due to pain. He takes a lot of painkillers (NSAIDs) and would like to get rid of the pain. He has now received physical therapy for more than a year, but the pain remains. Subacromial injections work very well, but the pain keeps coming back. The patient tells you his colleague had the same complaints, for which he was treated by subacromial decompression surgery. His colleague was able to return to work soon after surgery and the patient asks if this would be a possibility for him as well.

During physical examination you see antalgic restriction of shoulder movement and a painful arc is present. The empty can and Hawkins tests are both positive. The cross-chest test is negative. Additional imaging shows no changes compared to the previous year. The patient has smoked all his life (30 pack years) and is treated by the cardiologist for Angina Pectoris. The healthcare insurer reimburses both surgical and non-surgical management.

1. Would you perform subacromial decompression surgery in this patient?

- Yes
- No

2. What is your estimate of the likelihood of pain reduction from subacromial decompression surgery in this patient?

|  | 0 | 10 | 20 | 30 | 40 | 50 | 60 | 70 | 80 | 90 | 100 |
| --- | --- | --- | --- | --- | --- | --- | --- | --- | --- | --- | --- |

| I estimate the likelihood of pain reduction (%) | 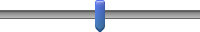 |
| --- | --- |

3. What is your estimate of the risk of complications from subacromial decompression surgery in this patient?

|  | 0 | 10 | 20 | 30 | 40 | 50 | 60 | 70 | 80 | 90 | 100 |
| --- | --- | --- | --- | --- | --- | --- | --- | --- | --- | --- | --- |

| I estimate the likelihood of complications (%) | 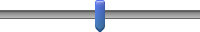 |
| --- | --- |

4. What are the most important factors influencing your decision to perform subacromial decompression surgery or not in this patient?

*Factors:*

- *Male, 51 years*
- *Painter*
- *>1 year complaints*
- *Progressive complaints*
- *Complaints limiting work/hobby/sleep*
- *Unilateral complaints*
- *Multiple visits at outpatient clinic*
- *Trauma in history*
- *Uses a lot of painkillers*
- *Range of motion limited*
- *Painful arc*
- *Specific tests: Hawkins positive, empty can positive, cross-chest negative*
- *Imaging: bursitis-like abnormalities, partial supraspinatus tendon rupture*
- *Non-surgical treatment better*
- *Other surgery better*
- *Surgery reimbursed*
- *Surgical treatment not indicated*
- *No effect of physical therapy on complaints*
- *Reduction in complaints after subacromial infiltration*
- *Patient asks about surgery*
- *Comorbidities of patient*

5. Please rank the factors that you have chosen where 1 indicates the most important factor.

1. …..
2. …..
3. …..
4. …..
5. …..

Clinical case 4

A 36-year-old woman has been experiencing pain complaints in the front of her right shoulder since 6 months. She is a professional volleyball player and the season has just ended. She mainly experiences pain symptoms when serving and smashing the ball. Despite her pain complaints, she has continued playing sports. This went reasonably well with painkillers and after a subacromial injection from the general practitioner. There has been no clear traumatic moment. She did go straight to the physical therapist, but physical therapy had no effect. Meanwhile the pain is such that she is impaired in performing daily activities and cannot play sports anymore. The last two weeks things seem to go slightly better (end of the season). She wants to be fit for the new season as soon as possible. Her physical therapist has contacted you and does not know what else he can do, he suggests surgery.

During physical examination, the range of motion of the right arm is slightly reduced compared to the left arm. There is a low painful arc and the Hawkins test is positive. The cross-chest test is negative. Additional imaging shows no abnormalities. The main healthcare insurer for your hospital has announced that they only reimburse 30% of surgical treatments for SAPS compared with the previous year. The departmental management has indicated that the 30% ceiling has almost been reached and that surgery possibly may not be reimbursed.

1. Would you perform subacromial decompression surgery in this patient?

- Yes
- No

2. What is your estimate of the likelihood of pain reduction from subacromial decompression surgery in this patient?

|  | 0 | 10 | 20 | 30 | 40 | 50 | 60 | 70 | 80 | 90 | 100 |
| --- | --- | --- | --- | --- | --- | --- | --- | --- | --- | --- | --- |

| I estimate the likelihood of pain reduction (%) | 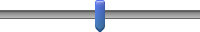 |
| --- | --- |

3. What is your estimate of the risk of complications from subacromial decompression surgery in this patient?

|  | 0 | 10 | 20 | 30 | 40 | 50 | 60 | 70 | 80 | 90 | 100 |
| --- | --- | --- | --- | --- | --- | --- | --- | --- | --- | --- | --- |

| I estimate the likelihood of complications (%) | 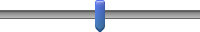 |
| --- | --- |

4. What are the most important factors influencing your decision to perform subacromial decompression surgery or not in this patient?

*Factors:*

- *Female, 36 years*
- *Professional volleyball player*
- *6 months complaints*
- *Less complaints after rest*
- *Complaints limiting work/sports/daily life*
- *Unilateral complaints*
- *First presentation on outpatient clinic*
- *No history of trauma*
- *Regular use painkillers*
- *Range of motion limited*
- *Painfull arc*
- *Specific tests: Hawkins positive, cross-chest negative*
- *No abnormalities on X-shoulder*
- *Imaging: no abnormalities*
- *Non-surgical treatment better*
- *Other surgery better*
- *Surgery may not be reimbursed*
- *Surgical treatment not indicated*
- *No effect of physical therapy on complaints*
- *Reduction in complaints after subacromial infiltration*
- *Suggestion surgery by physical therapist*
- *No explicit wish patient to have surgery*

5. Please rank the factors that you have chosen where 1 indicates the most important factor.

1. …..
2. …..
3. …..
4. …..
5. …..

**Part 4. Policy measures by healthcare insurer**

*During the previous clinical cases, it was described whether surgery would be reimbursed by a healthcare insurer. The withdrawal of reimbursement for specific treatments (a financial disincentive) aims to reduce the use of treatments that are considered to be low-value care. Financial disincentives are currently used by healthcare insurers for the treatment of SAPS.*

*From 2020 onwards, one specific healthcare insurer considers the surgical treatment of SAPS to be low-value care. Therefore, only 70% of these surgical procedures will be reimbursed in comparison to the preceding year.*

*The following questions relate to this financial disincentive by the specific healthcare insurer and its potential effect. Choose the most appropriate answer for each question.*

1. Are you aware of this financial policy measure for subacromial decompression surgery for subacromial pain syndrome?

- Yes, this concerns the following health care insurer …..
- Yes, but I don’t know which healthcare insurer.
- No

2. Would you be more reluctant to treat SAPS by performing subacromial decompression surgery if you were aware of a financial policy measure (regardless by which healthcare insurer)?

I do not weigh this at all in my decision I Weigh this considerably in my decision

|  | 1 | 2 | 3 | 4 | 5 | 6 | 7 |
| --- | --- | --- | --- | --- | --- | --- | --- |

|  | 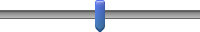 |
| --- | --- |

3. *The financial policy measure of the specific healthcare insurer states that they consider 80% of the surgical procedures for SAPS to be low-value care. Therefore, they will only reimburse 70% of these surgical procedures in comparison to the preceding year.*

Suppose that subacromial decompression surgery for SAPS would no longer be reimbursed in the future. To what extent would you still perform surgery on a patient with SAPS?

Never Often

|  | 1 | 2 | 3 | 4 | 5 | 6 | 7 |
| --- | --- | --- | --- | --- | --- | --- | --- |

|  | 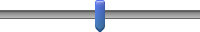 |
| --- | --- |

4. In such a situation (0% reimbursement for subacromial decompression surgery), what could be an important reason for you to still perform surgery on a patient with SAPS? [Fill in none if you do not perform surgery on patients with SAPS]

________________________________________________________________

5. Do you think there is sufficient scientific evidence to justify withdrawal of reimbursement by healthcare insurers for subacromial decompression surgery for SAPS?

Absolutely insufficient evidence Absolutely sufficient evidence

|  | 1 | 2 | 3 | 4 | 5 | 6 | 7 |
| --- | --- | --- | --- | --- | --- | --- | --- |

|  | 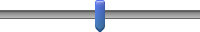 |
| --- | --- |

6. [Optional] Do you think that this type of policy measures (financial disincentive) are effective in reducing the use of subacromial decompression surgery for SAPS? Please substantiate.

________________________________________________________________

**End of survey**

Thank you very much for your time and completing the survey.

Any questions and/or comments can be posted below.

________________________________________________________________

________________________________________________________________

________________________________________________________________

________________________________________________________________

________________________________________________________________
