# Supplementary material for: Substantial Variation in Decision Making to Perform Subacromial Decompression Surgery for Subacromial Pain Syndrome Between Orthopaedic Shoulder Surgeons for Identical Clinical Scenarios: A Case-Vignette Study
Source: Arthrosc Sports Med Rehabil. 2023 Nov 11;5(6):100819. doi: 10.1016/j.asmr.2023.100819 (PMC10661501; doi:10.1016/j.asmr.2023.100819)
Supplement: Appendix Table 1 — Influence of Respondents’ Characteristics on Decision to Perform Surgery Across All Scenarios, Adjusted for Clustering Within Respondents and Sensitivity Analysis [file mmc3.docx]

**Appendix S2.**

**Results of univariate analysis. Influence of respondents’ characteristics on the decision to perform surgery across all scenarios, adjusted for clustering within respondents.**

|  | **OR (95CI)** | **P-value** |
| --- | --- | --- |
| **Sex*** | 1.305 (0.604-2.818) | 0.498 |
| **Age** | 1.007 (0.968-1.047) | 0.731 |
| **Function**** | 1.169 (0.511-2.674) | 0.712 |
| **Type Hospital***** | 1.503 (0.701-3.223) | 0.295 |
| **Experience** | 1.012 (0.966-1.061) | 0.608 |
| **SAPS patients per month** | 0.998(0.991-1.006) | 0.671 |
| * Reference is male ** Reference is orthopedic surgeon *** Reference is teaching hospital | | |

**Results of sensitivity analysis (i.e. without responses of orthopaedic residenst) compared to the initial analysis (data of sensitivity analysis is shown in red)**

**Probabilities of perceived benefit (i.e. pain reduction) and harms (i.e. complications) of subacromial decompression surgery for each clinical scenario, stratified by the decision to perform surgery.**

|  | **Clinical scenario** | | | | | | | |
| --- | --- | --- | --- | --- | --- | --- | --- | --- |
|  | **Scenario 1.**  **58-year-old construction worker** | | **Scenario 2.**  **48-year-old woman with previous subacromial decompression** | | **Scenario 3.**  **51-year-old painter** | | **Scenario 4. 36-year-old volleyball player** | |
| **Surgery** | **No (n=56) 13%**  **46 (13%)** | **Yes (n=8)**  **87%**  **6(87%)** | **No (n=47)**  **78%**  **39 (80%)** | **Yes (n=13)**  **22%**  **10 (20%)** | **No (n=43)**  **75%**  **35 (74%)** | **Yes (n=14)**  **25%**  **12 (26%)** | **No (n=55)**  **96%**  **45 (96%)** | **Yes (n=2)**  **4%**  **2 (4%)** |
| **Median probability of benefit (IQR)** | 25 (10-50)  23 (10-51) | 75 (70-88)  70 (65-83) | 29 (10-50)  22 (10-50) | 70 (50-81)  72 (50-84) | 20 (9-39)  19 (8-30) | 79 (50-81)  79 (55-82) | 15 (6-39)  10 (6-31) | 71 (70-71)  71 (70-71) |
| **Median probability of harm (IQR)** | 9 (4-17)  9 (4-14) | 9 (7-10)  4 (3-9) | 10 (5-19)  10 (5-19) | 5 (4-12)  5 (3-9) | 13 (8-20)  11 (8-20) | 8 (4-14)  8 (3-13) | 8 (3-15)  8 (3-17) | 3 (1-4)  3 (1-4) |

IQR = Interquartile Range.
